# Hydrophilic Polymers

**DOI:** 10.3390/polym11040693

**Published:** 2019-04-16

**Authors:** Bernhard V.K.J. Schmidt

**Affiliations:** 1 Max Planck Institute of Colloids and Interfaces, 14424 Potsdam, Germany; bernhard.schmidt@mpikg.mpg.de; Tel.: +49-331-567-9509; 2 School of Chemistry, University of Glasgow, Glasgow G12 8QQ, UK

Hydrophilic polymers are a major area of polymer research with prominent fields of application, e.g., in drug delivery [1], self-assembly [2,3], or catalysis [4]. As such, studies regarding hydrophilic polymers are an important part of polymer science. Especially investigations on structure-property relationships of hydrophilic polymers as well as interactions of hydrophilic polymers or the combination with biological entities are a key research area [5,6,7,8]. Here, synthetic polymer chemistry opens up new opportunities via novel water-soluble polymer types, architectures or combinations for advanced properties and future applications [9,10]. Hydrophilic polymers not only feature significant properties in the dissolved state, but also as crosslinked materials, namely in hydrogels. This class of materials presents various interesting properties and tailored shape and size, which is of major interest for biomedical applications as well [11]. 

A frequently asked question arises from the term “hydrophilic polymer” as there are several ways to define it. Here the reader is also referred to the review by Kressler, et al. that highlights this issue [12]. Notably, there is a broad variety of hydrophobic polymers that is not considered to be hydrophilic at all, e.g., poly(styrene) or poly(methyl methacrylate). Contrarily, there are various polymers considered to be hydrophilic, e.g., poly(acrylamide) or poly(ethylene glycol) (PEG). Nevertheless, the boundaries are not defined. Some polymers are hydrophilic but not water soluble and rather water-swellable, e.g., poly(2-hydroxyethyl methacrylate). Even for hydrophilic polymers solubility in water will be different depending on the polymer type, molecular weight and concentration. Thus, a classification on solubility alone is not sufficient. As such, a hydrophilic polymer might be considered as a polymer with favorable interactions with water leading to solubility or swellability. Of course, expanding the term to block copolymers or including stimuli response increases the difficulty of a classification drastically, e.g., some polymers are hydrophilic at specific pH but rather hydrophobic in other pH ranges or amphiphilic block copolymers uniting hydrophobic and hydrophilic character. There seems to be no clear answer to the issue of defining hydrophilic polymers but in the end no answer might be needed at all. A final classification would just introduce a virtual boundary in the field that might not be constructive at all.

In the present Special Issue, 11 primary articles as well as two reviews on hydrophilic polymers are collected that cover a broad range of topics, e.g., polymer characterization, recognition of hydrophilic polymers, self-assembly, hydrogels and microgels. A major component of polymer research is polymer characterization, which is a challenging task especially in the case of hydrophilic polymers. Schubert, et al. reported a fast method to identify alcohol end function impurities in PEG, which is a well-known hydrophilic polymer utilized in biomedical applications. [13] Reversed phase liquid chromatography on C18 derivatized monolithic silica rods was utilized to selectively elute PEG diol fraction next to methoxylated PEG with fractions of less than 1% in short measurement times.

A significant feature of several types of hydrophilic polymer is the ability to interact with biomacromolecules, e.g., with DNA or proteins [14,15]. An example of recognition with the protein concanavalin A was investigated by Wu, et al. [16]. Therefore, hyperbranched glycopolymers based on α-d-mannopyranose were synthesized from 2-(α-d-mannopyranoyloxy) ethyl methacrylate and *N*,*N*-methylene bisacrylamide via reversible addition fragmentation chain transfer (RAFT) polymerization. Subsequently, the lectin binding with concanavalin A was studied with respect to degree of branching and molecular mass of the hyperbranched polymer. It could be shown that the interaction increases with molecular mass and decreases with the degree of branching. Thus, the polymer structure could be correlated with a function, namely interaction with a biological entity.

Aggregation and self-assembly is one of the most striking abilities of hydrophilic polymers and has been a prominent part of the literature for several decades [17,18]. Notably, a distinction between the aggregation behavior of pure hydrophilic polymers and amphiphilic or even polyphilic polymers has to be made. This issue has been addressed by Kressler et al., who summarized the philicity concept in a review [12]. The review is a good way to get an insight into the spectrum of philicity of polymers, and the consequences for self-assembled structures in solution. Grimm and Schacher presented a dual stimuli responsive copolymer, namely poly(*N*-isopropylacrylamide-*co*-spiropyran acrylate) [19]. Both temperature and/or light response was studied in the solution and solid state showing a reversible switching from coil to globule state. Not only could the state of the copolymer be changed but also the cloud point temperature itself was affected by light irradiation. A cell-membrane inspired amphiphilic copolymer and its association behavior was described by Yusa and coworkers [20]. Copolymers were formed from hydrophilic 2-methacryloyloxyethyl phosphorylcholine (MPC) and hydrophobic *n*-dodecyl methacrylate via RAFT polymerization. In an aqueous environment, interpolymer aggregation was observed. It was found that the aggregate featured a hydrophilic MPC surface as no adsorption of bovine serum albumin was evident, which might be interesting for delivery applications.

A common class of polymer materials based on hydrophilic polymers is hydrogels that feature various significant properties, e.g., swelling, soft character and biocompatibility. Further, for hydrogels, applications in the biomedical field are a major emphasis, for example in tissue engineering or implants [21]. Our team described a combination of hydrophilic polymer self-assembly and hydrogel formation [22]. Therefore, the double hydrophilic block copolymer poly(*N*-vinylpyrrolidone)-*b*-poly(oligo ethylene glycol methacrylate) was combined with α-cyclodextrin (α-CD). The formation of a supramolecular complex between α-CD and the block copolymer in water led to the formation of supramolecular hydrogels with remarkable thermoresponsive behavior. Hence, the hydrogels showed thermoadaptive behavior, i.e., heating of the gels to different temperatures led to different mechanical properties after cooling to ambient temperature. Thus, the hydrogel “knows” its thermal history. As such CDs can be utilized for the gelation but they can be utilized for various other purposes in hydrogels as well, e.g., stimulus response or cargo loading. [23] Bian and coworkers utilized the kinetics of β-CD inclusion complex formation to obtain delayed crosslinking hydrogels [24]. Therefore, an amphiphilic polymer was synthesized from hydrophilic acrylamide, sodium acrylate and hydrophobic *N*-dodecylacrylamide. For the gelation an inclusion complex of β-CD and phenol was added that released phenol slowly to the environment via interaction with the dodecyl groups. Finally, the released phenol participated in a phenolic resin formation with formaldehyde to form the hydrogel. Thus, the formation of phenolic network crosslinking points was delayed via the inclusion complex.

A hydrogel based on an emulsion of lemon grass essential oil and alginate was described by Cuomo and coworkers [25]. To induce gelation, Ca^2+^ ions were slowly introduced into the emulsion from the Ca^2+^/EDTA complex via a pH change. The mechanical properties of the hydrogels were studied with respect to essential oil, alginate and Ca^2+^ concentration. Another polysaccharide hydrogel was presented by Thiele and coworkers, yet in this case microgels were targeted via microfluidics [26]. Therefore, hyaluronic acid or chitosan was combined with a PEG crosslinker and *tert*-butyl isocyanide. The crosslinking was performed via a Passerini three component reaction (with PEG dialdehyde) or an Ugi four component reaction (with PEG diamine and formaldehyde). The microfluidic approach allowed fabrication of mono-disperse microgels with sizes in the range of 70-100 µm. Finally, functional microgels were formed via addition of functional carboxylic acids in the Ugi crosslinking reaction, e.g., for the attachment of biotin. The functional microgels might be interesting for drug delivery, for example after loading of cargo in the gel and functionalization with targeting ligands.

A deep insight into the microstructure of PEG-based hydrogels was described by Lensen and coworkers [27]. Therefore, 8-arm star shaped PEG were crosslinked via two pathways: either via photo-initiated chain-growth polymerization or Michael addition step-growth polymerization. Finally, the obtained hydrogels were dried and the obtained structures analyzed with respect to crystallinity. Apparently, the size of crystalline domains is strongly affected by the polymerization process. Notably, this knowledge can be used to tailor the crystallization behavior in PEG-based hydrogels and finally the properties of the materials. A common way to improve the mechanical properties of hydrogels is reinforcement via the addition of nanoparticles [28]. Weiss and coworkers investigated the formation and mechanical properties of Laponite/cellulose-based hydrogels [29]. Therefore, Laponite was combined with silanized hydroxypropylmethylcellulose to form a crosslinked hydrogel. A significant reinforcement effect was observed compared to the Laponite-free hydrogel, i.e., an increase of storage modulus by an order of magnitude. Moreover, the hydrogel architecture was studied via fluorescent microsphere tracking analysis. Two domains were revealed as the hydrogel consists of a dense Laponite structures and loose aggregate areas in the surrounding.

Hydrophilic polymers - as mentioned before - have found significant applications in the biomedical field [1]. Hence, Zhao and coworkers summarized the recent developments of polymer-based nanomaterials for vaccines and drugs [30]. For example, natural, biosynthesized and chemically synthesized polymer-derived nanoparticles were described that were used as delivery carriers and vaccine adjuvants. In such a way properties like slow release, targeted delivery, alternative administration and delivery pathways were introduced. A class of hydrophilic polymers that has attracted attention recently is poly(peptoids) [31]. In particular, Barz and coworkers utilized poly(sarcosine) as a shielding agent for siRNA polyplexes [32]. Therefore, polyplexes of siRNA and a lipo-oligomer structure were formed. The lipo-oligomer contained two cholanic acids attached via a bioreducible disulfide linker to an oligoaminoamide backbone in T-shape configuration with azide-functionality. The azide function was utilized to graft alkyne functional poly(sarcosine) on the polyplex units. In the subsequent step, the shielding ability was probed with biophysical assays and bioimaging in vivo. Indeed, a significantly longer blood circulation time was observed similar to the gold standard PEG.

Overall, the presented collection of research articles and reviews surrounding the topic of hydrophilic polymers shows the broad variety of research that is being performed in polymer science. It may serve as a reference for readers interested in the topic to obtain an insight into the field. As Editor of this Special Issue, I want to take the chance to acknowledge the authors and reviewers for their valuable contribution, as well as the Editorial team for the smooth organization.
